# The role of daily physical activity and nutritional status on bone turnover in cystic fibrosis: a cross-sectional study

**DOI:** 10.1590/bjpt-rbf.2014.0154

**Published:** 2016-03-18

**Authors:** Sergio Tejero, Pilar Cejudo, E. Quintana-Gallego, Borja Sañudo, A. Oliva-Pascual-Vaca

**Affiliations:** 1 Departement of Phisical Therapy, Universidad de Sevilla (US), Sevilla, Spain; 2 Department of Trauma and Orthopedic Surgery, HU Virgen del Rocío, Sevilla, Spain

**Keywords:** cystic fibrosis, physical therapy, rehabilitation, nutritional status, bone remodeling, bone mineral density

## Abstract

**Background:**

Nutritional status and daily physical activity (PA) may be an excellent tool for the maintenance of bone health in patients with cystic fibrosis (CF).

**Objective:**

To evaluate the relationship between nutritional status, daily physical activity and bone turnover in cystic fibrosis patients.

**Method:**

A cross-sectional study of adolescent and adult patients diagnosed with clinically stable cystic fibrosis was conducted. Total body, femoral neck, and lumbar spine bone mineral density (BMD) were determined by dual energy X-ray absorptiometry and bone metabolism markers ALP, P1NP, PICP, and ß-CrossLaps. PA monitoring was assessed for 5 consecutive days using a portable device. Exercise capacity was also determined. Serum 25-hydroxyvitamin D and vitamin K were also determined in all participants.

**Results:**

Fifty patients (median age: 24.4 years; range: 16-46) were included. BMI had positive correlation with all BMD parameters, with Spearman’s coefficients ranging from 0.31 to 0.47. Total hip bone mineral density and femoral neck BMD had positive correlation with the daily time spent on moderate PA (>4.8 metabolic equivalent-minutes/day; r=0.74, p<0.001 and r=0.72 p<0.001 respectively), daily time spent on vigorous PA (>7.2 metabolic equivalent-minutes/day; r=0.45 p<0.001), body mass index (r=0.44, p=0.001), and muscle mass in limbs (r=0.41, p=0.004). Levels of carboxy-terminal propeptide of type 1 collagen were positively associated with the daily time spent on moderate (r=0.33 p=0.023) and vigorous PA (r=0.53, p<0.001).

**Conclusions:**

BMI and the daily time spent on moderate PA were found to be correlated with femoral neck BMD in CF patients. The association between daily PA and biochemical markers of bone formation suggests that the level of daily PA may be linked to bone health in this patient group. Further research is needed to confirm these findings.

## Bullet Points

- Physical therapy in cystic fibrosis patients is correlated with bone health.
- Bone health can improve with physical activity.

## Introduction

Thanks to advances in the treatment and care of patients with cystic fibrosis (CF), the life expectancy^1^ of these patients has increased. However, this fact has not resulted in quality of life because different comorbidities have been observed in adulthood. Disturbances in bone metabolism, particularly significant reductions in bone mineral density (BMD), are recognized as additional and serious complications in these CF patients, with prevalence ranging from 40% to 70%^2^ ^,^ ^3^. The low BMD found in these patients is multifactorial and many factors have been suggested as explanation^4^ ^-^ ^10^. One of these explanations is based on intestinal malabsorption, with vitamin D deficiency caused by exocrine pancreatic insufficiency^4^. Evidence suggests that the absorption of vitamins D and K may not be sufficient to ensure proper skeletal mineralization in CF adolescents^5^. Another bone metabolic disorder found in CF patients is imbalance in bone turnover, with decreased bone formation as the predominant change and increased bone resorption in infective exacerbation or severe lung disease^6^ ^-^ ^8^. Moreover, severe lung disease also affects bone mass. Forced expiratory volume in one second (FEV_1_) expressed as the percentage of the predicted value is a robust marker for lung disease and helps to explain the variability of BMD^9^. This correlation is explained in part because severe lung disease adversely affects exercise capacity. When monitoring daily physical activity (PA), it was noted that the most active patients have greatest bone mass, confirmed by exercise capacity test^10^. Nevertheless, there is no confirmation of whether bone turnover has a relationship with PA and its intensity or with nutritional status. This study was designed to evaluate the relationship between daily level of PA, nutritional status, and bone parameters (BMD and biomarkers of bone turnover) in a sample of adolescents and adults with CF.

## Method

### Participants and study design

We conducted a cross-sectional study that included patients over 16 years of age diagnosed with CF and attending our Cystic Fibrosis Unit. All participants exhibited high sweat chloride levels (>60 mmol/L), had characteristic clinical features of CF, and their diagnoses were confirmed by repeated genetic analysis. Patients who had undergone lung transplantation and/or had a recent acute exacerbation requiring treatment with antibiotics (within 6 weeks prior to the study) were excluded. The Ethics Committee of the Virgen del Rocío Hospital, Sevilla, Spain approved this study (approval number 14/2008) and written informed consent was obtained from all participants.

### Maximal cardiopulmonary exercise testing

In order to evaluate the tolerance to exercise, maximal cardiopulmonary exercise testing was performed with a MasterScreen CPX cycle ergometer model Via Sprint 150 (ViaSys Healthcare, Hoechberg, Germany), as previously described^10^ and according to the international standard^11^. The test is a symptom-limited exercise and involves measurements of respiratory oxygen uptake (VO_2_), carbon dioxide production (VCO_2_), and ventilatory measures. It was performed with electrocardiographic, heart rate, and pulse oximetry monitoring. All measurements were integrated into the cycle ergometer device and evaluated simultaneously during the test. On completion of the exercise test, heart rate, blood pressure, leg fatigue, chest pain, and dyspnea were assessed using the modified Borg scale.

### Physical activity monitoring

Physical activity was measured in all participants on five consecutive days using a portable physical activity and lifestyle monitor (SenseWear® Armband, BodyMedia Inc., Pittsburgh, PA, USA), which estimates a person’s rest and exercise energy expenditure. The accelerometer was positioned on the right brachial triceps and was only removed for showering^12^. The records were considered valid if the mean wearing time was ≥600 min/day in the 5-day period and at least one of the five days landed on a weekend. The use of this device has been validated against doubly labeled water^13^. The time (in minutes) spent with an energy expenditure of >3 metabolic equivalent-minutes/day (METs) was considered “mild” activity, time spent at >4.8 METs was considered “moderate” activity, and when energy expenditure was of >7.2 METs, it was considered “vigorous” activity, as suggested by the U.S. Department of Health and Human Services^14^. The average number of daily steps at each intensity level was calculated in all participants.

### Nutritional assessment

The values for lean muscle mass and fat mass were assessed in the extremities by dual energy X-ray absorptiometry^15^ (DXA). Body mass index, serum albumin, and serum levels of vitamins D and K were determined in all participants. For biochemical assays, blood samples were drawn in the morning after an overnight fast. Concentrations of 25-hydroxyvitamin D (25OHD) were determined by high-performance liquid chromatography (HPLC) on an Agilent 1100 HPLC system (Agilent Technologies Inc., Santa Clara, CA, USA). Plasma vitamin K concentrations were assessed by means of HPLC.

### Bone mineral density and bone turnover biomarkers

Total body, femoral neck, and lumbar spine (L1-L4) BMD were determined by dual-energy X-ray absorptiometry (Hologic QDR X-ray 4500W Bone Densitometer). DXA results were expressed in absolute BMD units (grams per square centimeter), T-scores, and Z-scores. The T-score was defined as the number of standard deviations above or below the mean for a healthy 20-year-old adult of the same sex and ethnicity as the patient. The Z-score was defined as the number of standard deviations a patient's BMD differs from the average BMD of their age, sex, and ethnicity^16^.

Serum levels of β-CrossLaps and amino-terminal propeptide of type 1 collagen (P1NP) were measured using an electrochemiluminescence method on a Cobas e411 analyzer (Roche Diagnostics, Indianapolis, IN, USA). Bone-specific alkaline phosphatase (ALP) and serum levels of carboxy-terminal propeptide of type 1 collagen (P1CP) were assayed using ELISA (Quidel Corporation, San Diego, CA, USA) on an automated TRITURUS processor (Grifols International S.A., Barcelona, Spain).

### Statistical analysis

The simple size calculation was estimated using the Dixon and Mansey formula^17^. This sample size was calculated using a power of 80% and a type I error of 5%.

Continuous data were expressed as medians and interquartile ranges (Q3- Q1) and categorical data were expressed with relative frequencies. We performed a Mann-Whitney U test and Chi-Square test for comparisons between sex groups. The associations between functional and structural parameters were tested using the non-parametric Rho Spearman correlation. Spearman's rank correlation can be interpreted in terms of the amount of mutual information between two variables. An observed value of ρ is significantly different from zero (r will always maintain −1≤r≤1). We calculated the probability that it would be greater than or equal to the observed r, given the null hypothesis, by using a permutation test. An advantage of this approach is that it automatically takes into account the number of tied data values in the sample and the way they are treated in computing the rank correlation. All tests were performed using 0.2 and 0.05 as the probability of a Type II error and probability of a Type I error, respectively.

All statistical calculations were performed using the SPSS 16.0 statistical software package (SPSS Inc., Chicago, IL, USA). Two-tailed p<0.05 were considered statistically significant.

## Results

We included 50 patients in our sample (23 males and 27 females) with a median age 24.4 (interquartile range - IQR=5.8) years. A total of 48 patients (96%) were undergoing oral corticosteroid treatment. Two of the patients (4%) had been diagnosed with allergic bronchopulmonary aspergillosis (ABPA) and were being treated intermittently with prednisone (10 mg/day per os) for the last three years, and 23 patients (96%) had also been receiving inhaled corticosteroids (budesonide) at doses of 200-400 μg administered twice a day. All patients were prescribed vitamin supplementation (Vitamin A 1.50 mg, Vitamin D 0.01 mg, and Vitamin E 200 mg). There was no documented secondary hepatopathy in any patient.

The male patients showed more wattage, 130 vs 100 (p=0.001) and spent more time on a moderate activity 24 vs 11.6 (p=0.045). There was no difference in the remaining parameters between sex groups. The general characteristics of the study participants are shown in Table 1.

**Table 1:** General characteristics of patients with cystic fibrosis stratified according to sex.

	**Total Cohort (N=50)**	**Males** **(n=23)**	**Females** **(n=27)**	**p**
**Demographic data**				
Age years (IQR)	24.5 (19-28)	25 (19-30)	24 (20-27)	0.59
Corticosteroids	48 (96)	22 (95.6)	26 (96.3)	1.00
BMI Kg/m^2^ (IQR)	20.5 (18.8-21.9)	20.6 (18.9-22.8)	19.9 (18.7-21.6)	0.30
ΔF508 mutation	33 (66.0)	16 (69.6)	17 (63.0)	0.62
ΔF508 homocigosis	15 (30.0)	8 (34.8)	7 (25.9)	0.50
ΔF508 heterocigosis	18 (36.0)	8 (34.8)	10 (37.0)	0.87
R334W mutation	10 (20.0)	6 (26.1)	4 (14.8)	0.32
Exocrine pancreatic insufficiency	36 (72.0)	15 (65.2)	21 (77.8)	0.32

**Exercise tolerance**				
FEV_1%_ predicted (IQR)	60.3 (35.2-80.9)	47.4 (35.2-80.8)	60.7 (32.6-82.6)	1.00
FVC % predicted (IQR)	78.0 (59.3-95.3)	77 (59.3-93.5)	78.6 (58.1-99.9)	0.90
VO_2_max% (IQR)	66.5 (58.0-81.5)	65 (58-75)	72 (60.9-89.0)	0.22
Wmax (IQR)	110 (80-130)	130 (110-150)	100 (80-110)	**0.001**
6MWT distance meters (IQR)	640 (582-679)	647 (620-695)	615 (562-675)	**0.05**

**Daily physical activity**				
Device wearing min/day (IQR)	618 (598-637)	614 (598-643)	622 (589-634)	0.63
PA >3 METs min/day (IQR)	152 (107-231)	154 (117-239)	121 (97-230)	0.37
PA >4.8 METs min/day (IQR)	11.6 (5.5-35.4)	24 (6.25-56.55)	10.2 (3.5-16.6)	**0.04**
PA >7.2METs min/day (IQR)	0.5 (0-2.2)	0.75 (0.25-2.0)	0.16 (0-1.12)	0.05
Steps/day (IQR)	8,361 (5,662-10,322)	8,859 (5,676-12,472)	7,972 (5,294-9,660)	0.22

**Osteoporosis**				
Z score <1 SD (%)	25 (51.0)	12 (52.2)	13 (48.1)	0.45
Z score <2 SD (%)	2 (4)	2 (8.7)	0 (0)	0.22

IQR: interquartile range; BMI: body mass index; FEV_1_: forced expiratory volume in one second; FVC: forced vital capacity; VO_2_max% theoretical: maximal oxygen consumption; Wmax (watts): maximal power output; PA: physical activity; METs: metabolic equivalents (minutes/day). Data given as median (IQR).

### Nutritional status, bone mineral density, and markers

Body mass index (BMI) showed a positive correlation with all of the BMD parameters, with Spearman’s coefficient ranging from 0.31 to 0.47. Muscle mass had a positive relationsip with all markers ranging from 0.48 to 0.43 for bone formation markers and 0.33 for serum levels of β-CrossLaps. In contrast, the percentage of fat mass had a negative correlation with the formation and resorption markers, ranging from –0.57 to –0.34. A positive correlation was found between 25-hydroxyvitamin D and whole-body Z-scores (r=0.34, p=0.03).

### Daily physical activity, bone mineral density, and markers

Wattage showed a positive correlation with all BMD parameters. Spearman’s coefficient, ranging from 0.30 to 0.49, also showed a weak positive correlation with ALP (r=0.29 p=0.04).

**Figure 1:**
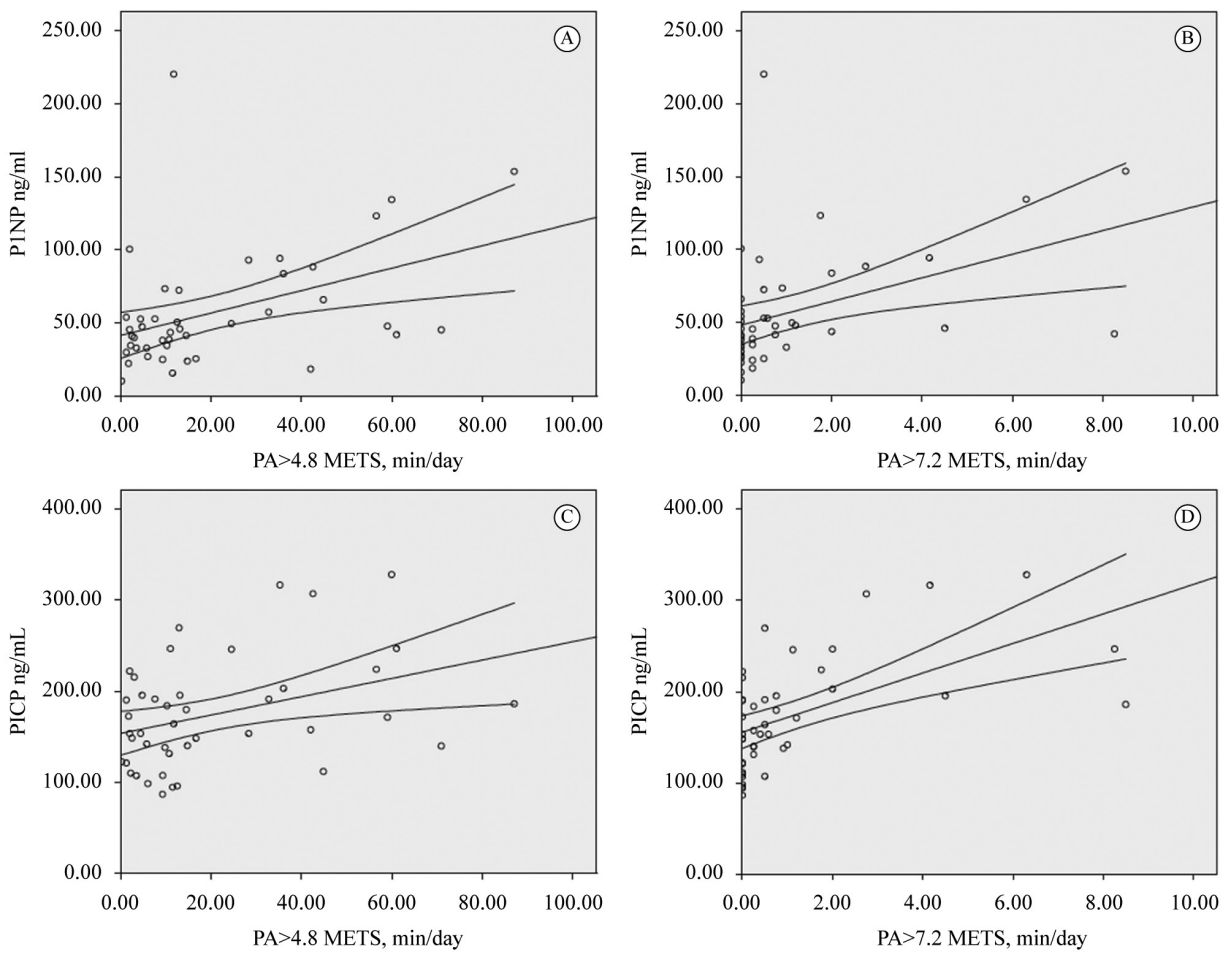
Correlation beetween bone formation markers and daily physical activity. (A) PA>4.8 METs min/day versus P1NP ng/mL, r=0.35 p=0.013; (B) PA>7.2 METs min/day versus P1NP ng/mL, r=0.42 p=0.003; (C) PA>4.8 METs min/day versus PICP ng/mL, r=0.32 p=0.023; (D) PA>7.2 METs min/day versus PICP ng/mL, r=0.53 p<0.001.

All variables of daily physical activity (PA) had a positive correlation with at least two BMD parameters. The strongest correlation was found between the average number of minutes spent on moderate daily PA (>4.8 METs) and hip BMD (r=0.74 p<0.001) (Table 2). Remodeling markers only had a positive correlation with moderate and vigorous daily PA with a higher coefficient in vigorous activity and formation markers. The relationship of bone formation markers with 4.8 METs and 7.2 METs is presented in Figure 1.

**Table 2:** Spearman’s correlations of exercise tolerance parameters, daily physical activity, and nutritional parameters with bone mineral density and bone turnover biomarkers in adult CF patients.

	**BMD**	**r**	**p**	**Bone Turnover** **Biomarkers**	**r**	**p**
**EXERCISE TOLERANCE AND DAILY PHYSICAL ACTIVITY PARAMETERS**						
PA >3 METs	Lumbar spine Total hip Femoral neck Whole body	0.17 **0.36** **0.28** **0.26**	0.254 **0.01** **0.048** **0.047**	P1NP PICP ALP ß-CrossLaps	0.13 0.18 -0.01 0.13	0.366 0.213 0.927 0.360
PA >4.8 METs	Lumbar spine Total hip Femoral neck Whole body	**0.58** **0.74** **0.72** 0.24	**<0.001** **<0.001** **<0.001** 0.098	P1NP PICP ALP ß-CrossLaps	**0.35** **0.32** **0.29** 0.16	**0.013** **0.023** **0.043** 0.266
PA >7.2 METs	Lumbar spine Total hip Femoral neck Whole body	0.20 **0.49** **0.34** 0.21	0.168 **<0.001** **0.016** 0.149	P1NP PICP ALP ß-CrossLaps	**0.42** **0.53** **0.36** **0.30**	**0.003** **<0.001** **0.011** **0.037**

**NUTRITIONAL PARAMETERS**						
BMI (Kg/m^2^)	Lumbar spine Total hip Femoral neck Whole body	**0.35** **0.44** **0.31** **0.48**	**0.015** **0.001** **0.028** **0.001**	P1NP PICP ALP ß-CrossLaps	0.10 -0.13 0 -0.04	0.480 0.369 0.999 0.804
Muscle mass (g)	Lumbar spine Total hip Femoral neck Whole body	0.03 **0.41** 0.16 0.16	0.819 **0.004** 0.275 0.272	P1NP PICP ALP ß-CrossLaps	**0.48** **0.49** **0.43** **0.33**	**<0.001** **<0.001** **0.002** **0.022**
Fat mass (%)	Lumbar spine Total hip Femoral neck Whole body	0.55 -0.25 -0.03 -0.00	0.711 0.087 0.837 0.991	P1NP PICP ALP ß-CrossLaps	**-0.43** **-0.58** **-0.37** **-0.34**	**0.002** **<0.001** **0.010** **0.017**
25OHD (nmol/L)	Lumbar spine Total hip Femoral neck Whole body	0.14 0.21 0.30 **0.34**	0.324 0.150 0.057 **0.034**	P1NP PICP ALP ß-CrossLaps	0.06 -0.15 0.07 0.02	0.672 0.313 0.645 0.896
VITAMIN K (μg/L)	Lumbar spine Total hip Femoral neck Whole body	0.01 0.00 -0.04 0.07	0.940 0.991 0.778 0.651	P1NP PICP ALP ß-CrossLaps	-0.09 -0.14 0.07 0.04	0.574 0.319 0.645 0.805

BMD: bone mineral density; METs: metabolic equivalents (minutes/day); P1NP: amino-terminal propeptide of type 1 collagen; P1CP: carboxy-terminal propeptide of type 1 collagen; ALP: bone-specific alkaline phosphatase; BMI: body mass index; 25OHD: 25-hydroxyvitamin D.

## Discussion

The foundations for lifetime bone health are established from infancy to adolescence and require adequate nutrition, body mass, PA, and hormone production^18^. CF compromises the gains during puberty when growth and mineral accrual are most rapid^19^. The values of BMD found in our CF patients are consistent with previous findings^2^. Our results show that patients diagnosed with CF with high levels of daily PA have higher BMD values and increased serum levels of bone formation markers. The bone remodeling markers in our sample were significantly different from the reference values of the normal adult population.

Routine screening for reduced BMD using dual energy X-ray absorptiometry (DXA) scans is recommended as detailed in published guidelines^2^ ^,^ ^20^. The recommendation is for this procedure to be performed every 5 years, 2 years, or 1 year, depending on Z-score values in young adults. Bone loss has been observed in several longitudinal studies in young adults with CF^21^ ^,^ ^22^ reporting annualized losses in BMD of 0.5 to 2.1%. This significant loss requires a more thorough investigation. The positive correlation found between BMI and the BMD values was in agreement with several previous studies^9^ ^,^ ^23^ and suggests that improving vigilance in the periods between DXA scans could result in an alternative marker.

Concerning vitamins, the normal rate of 25OHD in these patients was 20-40 ng/mL. We only found a weak positive relationship between 25OHD and BMD values, although all patients had vitamin D supplementation. Recent meta-analyses show that vitamin D supplementation increases the levels of vitamin D in the blood but does not improve BMD values^24^. This suggests that recommended vitamin supplementation is useful but insufficient for the maintenance of bone health in these patients^2^.

PA and exercise training play an important role in the clinical management of patients with cystic fibrosis (CF). Exercise training is more common and recognized as an essential part of rehabilitation programs and overall CF care^2^ ^,^ ^20^. However, exercise remains underutilized and not always incorporated into the CF management routine.

Our results suggest that there is no correlation of turnover markers in PA with low intensity. However, when daily physical activity is moderate (>4.8 METS) or vigorous (>7.2 METS), they are significantly correlated with circulating levels of bone formation markers. The present study is the first to demonstrate that vigorous daily PA may have a correlation with the rate of bone remodeling by favoring bone formation in adolescent and young adult CF patients. These findings suggest that the intensity of the exercise can have an influence on the bone turnover of CF patients and therefore should be controlled in the exercise prescriptions to improve bone health.

This is suggested in a review of weight-bearing exercise^25^, which states that exercise prescriptions should generate bone strains that are not only of a sufficiently high magnitude, but also unusually distributed and high rate.

Published data on the amount of time spent on PA among patients with CF are limited. Although Troosters et al.^12^ have previously described the use of accelerometers to monitor daily PA in this population group, they did not focus the possible effects on BMD nor on bone remodeling markers. In contrast, other authors have established models in which a patient’s maximal oxygen consumption (VO_2_max) could be used as a BMD predictor^26^ ^,^ ^27^.

There are some limitations to the present study. First, results on bone turnover markers in CF patients should be interpreted with caution. In particular, levels of bone turnover markers may be altered in a specific subset of CF patients with impaired liver function. In addition, alterations in BMI, use of steroids, changes in lifestyle, and other variables may have confounded the results of bone turnover markers. The cross-sectional nature of our study means that the effect of exercise on bone metabolism was collected retrospectively and there was no control of vitamin supplementation adherence. Nevertheless, it should be noted that exercise is unlikely to result in short-term changes in bone turnover markers^28^. Future longitudinal studies with long follow-up periods are needed to confirm and expand our findings.

Despite these limitations, BMI and daily time spent on moderate physical activity were found to be correlated with femoral neck BMD in CF patients. The correlation between daily PA and biochemical markers of bone formation suggests that exercise is linked to bone health in this patient group. Further longitudinal studies are needed in order to confirm these findings.

## Conclusion

BMI and daily time spent on moderate PA were found to be correlated with femoral neck BMD in CF patients. The correlation between daily PA and biochemical bone formation markers suggests that moderate and vigorous levels of PA may improve bone health in this population. Further research is needed in order to confirm these findings.
